# Draft Genome Sequence of an Aeromonas salmonicida subsp. *salmonicida* Strain from the Canadian Pacific Coast Bearing a Variant of pRAS1

**DOI:** 10.1128/MRA.00291-21

**Published:** 2021-05-06

**Authors:** Kevin C. Vaillancourt, Valérie E. Paquet, Antony T. Vincent, Anna Schneider, Christy Thompson, Mathilde Laurent, Michel Frenette, Steve J. Charette

**Affiliations:** a Institut de Biologie Intégrative et des Systèmes (IBIS), Université Laval, Quebec City, QC, Canada; b Département de Biochimie, de Microbiologie et de Bio-informatique, Faculté des Sciences et de Génie, Université Laval, Quebec City, QC, Canada; c Centre de Recherche de l’Institut Universitaire de Cardiologie et de Pneumologie de Québec (IUCPQ), Quebec City, QC, Canada; d Département des Sciences Animales, Faculté des Sciences de l’Agriculture et de l’Alimentation, Université Laval, Quebec City, QC, Canada; e Fish Health Diagnostics, Fisheries and Oceans Canada, Nanaimo, BC, Canada; f Groupe de Recherche en Écologie Buccale (GREB), Faculté de Médecine Dentaire, Université Laval, Quebec City, QC, Canada; University of Delaware

## Abstract

The genome sequencing of Aeromonas salmonicida subspecies *salmonicida* strain 2004-072 revealed a plasmid bearing a region carrying antibiotic resistance genes very similar to the one found in the plasmid pRAS1, an IncU family plasmid. This new plasmid was named pRAS1b.

## ANNOUNCEMENT

The Gram-negative bacterium Aeromonas salmonicida subsp. *salmonicida* is a major fish pathogen (1). This bacterium harbors a very diverse plasmidome (2) including a wide variety of plasmids found in Canadian isolates carrying antibiotic resistance genes (3–7).

In order to have a broader perspective of the genomic diversity among *A. salmonicida* subsp. *salmonicida* strains in Canada, we present the draft genome sequence of strain 2004-072, isolated in 2004 from a juvenile Atlantic salmon held at the Pacific Biological Station in Nanaimo, British Columbia, Canada. The exact origin of the fish is unknown. Samples from the fish were plated onto tryptic soy agar (TSA), and a brown-pigmented colony was isolated and initially identified using an API 20NE kit (bioMérieux, Saint-Laurent, QC, Canada).

The bacterial isolate was recovered from the frozen stock at −80°C and plated onto TSA and incubated at 18°C for 48 h. The total genomic DNA of strain 2004-072 was extracted using DNeasy blood and tissue kits from one colony grown in liquid medium (Qiagen, Montreal, QC, Canada) as previously described (8). Sequencing libraries were prepared from purified bacterial DNA using the Nextera XT DNA library preparation kit, and the sequencing was performed using a MiSeq instrument system (Illumina, San Diego, CA, USA) that generates paired reads of 300 bp (2 × 300 bp) at the Plateforme d’Analyse Génomique of the Institut de Biologie Intégrative et des Systèmes (Université Laval, Quebec City, QC, Canada). The resulting sequencing reads were analyzed using FastQC version 0.11.9 (https://www.bioinformatics.babraham.ac.uk/projects/fastqc/) and assembled using SKESA version 2.3.0 (9). The final assembly size was 4,751,504 bp, with 117 contigs, an *N*_50_ value of 115,043 bp, and a GC content of 58.48%. The genome sequence contains 4,419 coding DNA sequences (CDS) with a coverage of 80× and 1,459,598 reads. Default parameters were used for all bioinformatics tools except where otherwise noted.

The genome of the 2004-072 strain presents classical features of *A. salmonicida* subsp. *salmonicida* with plasmids pAsa1, pAsa2, pAsa3, and pAsal1 and the large plasmid pAsa5 bearing the type three secretion system locus (10–12). No *AsaGEI*, a group of genomic islands, was detected in this genome (13).

**FIG 1:**
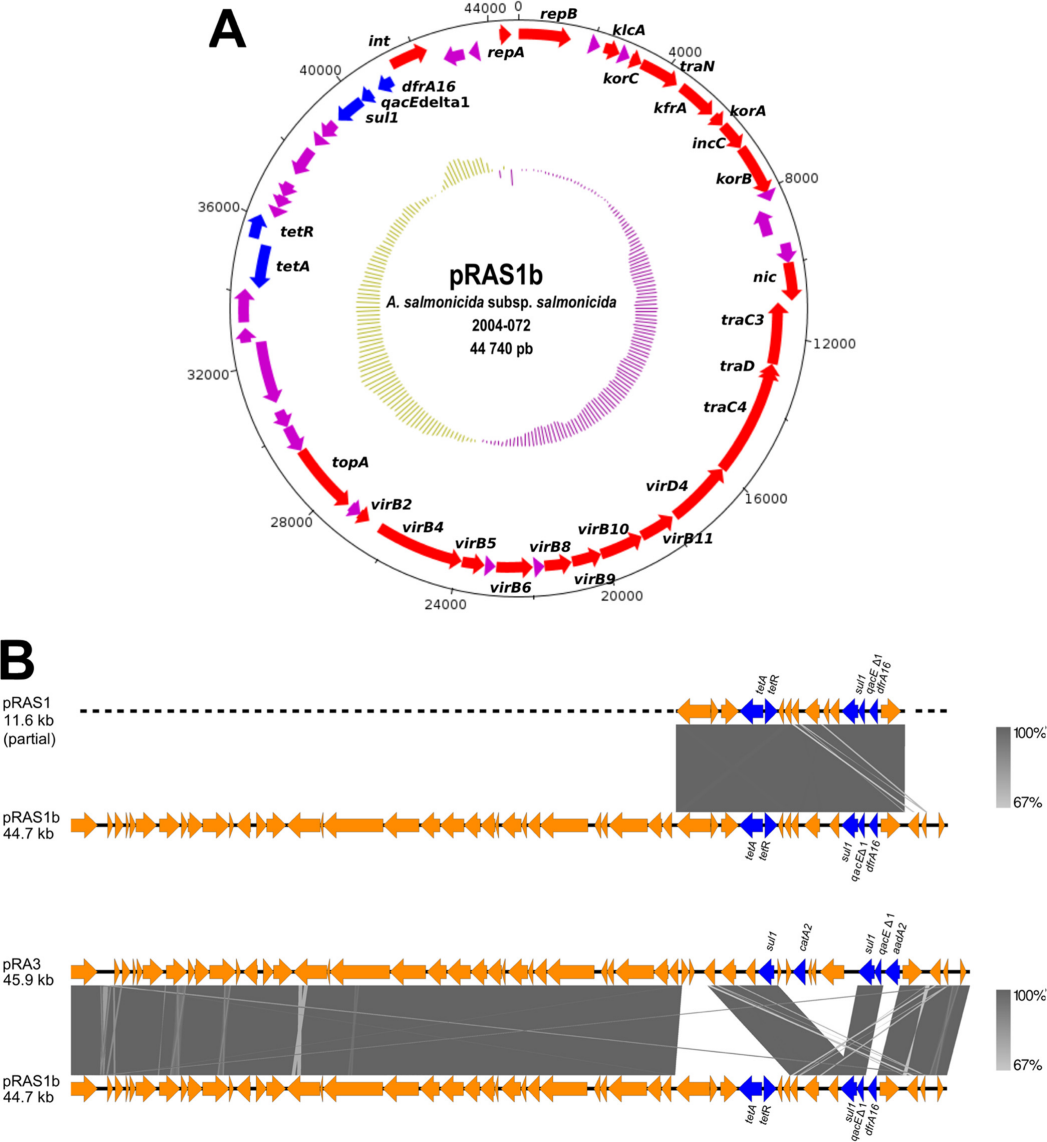
The plasmid pRAS1b. (A) The map of pRAS1b was produced with the DNAPlotter software (16). The red genes code for known proteins, the blue genes code for antibiotic resistance proteins, and the purple genes code for hypothetical proteins. The inner circle corresponds to the GC skew. (B) Similarities between pRAS1b, pRAS1, and pRA3. The gray areas represent similar or identical segments in the sequences of the plasmids compared. The blue arrows represent the antibiotic resistance genes. This part of the figure was made using Easyfig software (17).

The most notable element present in this genome is a 44.7-kb plasmid. A section of about 11.6 kb of this plasmid is highly similar to the class 1 integron and Tn*1721* transposon sequences of the pRAS1 plasmid (GenBank accession number AJ517790.2). The plasmid bears antibiotic resistance genes; the *dfr16* (trimethoprim) and *sul1* (sulfonamide) genes are found in the integron, while the *tetA* (tetracycline) gene is located in the transposon (14). The new plasmid was consequently named pRAS1b (Fig. 1A). Only the integron of pRAS1 was available in public databases (Fig. 1B). By sequencing pRAS1b, it is now possible to have access to the backbone sequence of this group of plasmids which share a highly conserved backbone with other IncU plasmids, such as pRA3, which is considered the reference for IncU plasmids found in the *Aeromonas* genus (Fig. 1B) (15). The sequence of pRAS1b was circularized by PCR and amplicon sequencing using the following primers: 5′-TGATATG GACAGCCACAAATG-3′ and 5′-CTTCACCGATCACGTCTTTG-3′.

### Data availability.

The genome sequence of strain 2004-072 has been deposited in DDBJ/ENA/GenBank under the following accession and BioSample numbers, respectively: JAFBAV000000000 and SAMN17709026. The raw sequencing reads have been deposited in the SRA (number SRR13748121). The pRAS1b closed sequence has been also deposited in DDBJ/ENA/GenBank (accession number MW033206).
